# Rapid peptide analysis in dried bloodspots to identify novel markers for newborn screening for congenital hypothyroidism

**DOI:** 10.1038/s41598-026-42578-w

**Published:** 2026-03-10

**Authors:** Jaranee Phoungphosop, Teerakul Arpornsuwan, Janthima Jaresitthikunchai, Narumon Phaonakrop, Siriwan Thaisakul, Penpan Thongngao, Piyanan Thiplakorn, Sittiruk Roytrakul, Piamnukul Krasao

**Affiliations:** 1 Department of Medical Sciences, Medical Life Sciences Institute, Nonthaburi, 11000 Thailand; 2 National Center for Genetic Engineering and Biotechnology, National Science and Technology for Development Agency, Klong Luang, Pathumthani, 12120 Thailand; 3 Department of Medical Technology, College of Allied Health Sciences, Rattana Bundit University, Pathumthani, 12160 Thailand

**Keywords:** Congenital hypothyroidism, MALDI-TOF MS, peptide barcode, peptidome, Biochemistry, Biomarkers, Diseases, Medical research

## Abstract

Early diagnosis of Congenital Hypothyroidism (CH) is critical to prevent irreversible neurodevelopmental damage. However, current TSH-based newborn screening using Dried Blood Spots (DBS) is limited by factors that lead to false-positive and false-negative results, necessitating the development of alternative methods. Matrix-Assisted Laser Desorption Ionization Time-of-Flight Mass Spectrometry (MALDI-TOF MS) as a high-throughput solution was investigated. This study presents a novel diagnostic approach utilizing DBS peptide barcoding, which was supported by comprehensive LC-MS/MS and network analysis. Peptide profiles showed a marked and reproducible difference between groups. MALDI-TOF MS identified a unique signature, including six dominant peptides specific to the CH-positive group. Subsequent analysis identified 37 candidate peptides, with network analysis linking 12 key proteins to established CH-related agents (e.g., thyroxine, TSHR). The MALDI-TOF MS peptidomic approach is validated as a robust, rapid, and cost-effective alternative for CH screening. This methodology represents a significant advance toward overcoming the limitations of current TSH assays and establishing a clinically translatable biomarker panel for improved personalized diagnosis of CH.

## Introduction

Congenital hypothyroidism (CH), a condition characterized by thyroid hormone deficiency at birth, can severely impair physical and neurological development, potentially leading to intellectual disability if left untreated^1^. Early diagnosis and intervention within the first 2–4 weeks of life are crucial for effective prevention and treatment^2,3^, highlighting the importance of newborn screening programs. CH affects approximately 1 in 3,000–4,000 newborns^2,4–6^.

Since 1996, Thailand has maintained a nationwide neonatal CH screening program through the Department of Medical Sciences, Ministry of Public Health^7^. Dried blood spot (DBS) samples, collected from heel pricks or dorsal hand veins of infants after 48 h of life, are analyzed using an in-house sandwich enzyme-linked immunosorbent assay (ELISA) to measure thyroid-stimulating hormone (TSH) levels. A TSH concentration ≥ 25 mU/L in DBS is considered a presumptive positive, requiring confirmatory testing of serum TSH and free thyroxine (FT4). A CH diagnosis is confirmed when serum TSH exceeds 11 mU/L and FT4 is below 0.83 ng/dL^8^.

Several factors can influence TSH levels in neonatal DBS and potentially lead to misdiagnosis. These include blood collection within 48 h of birth^9^, use of iodine-containing disinfectants during delivery^10,11^, maternal iodine deficiency, and double spotting of blood on filter paper^12^, all of which can contribute to falsely elevated TSH and false-positive results. Conversely, preterm or low-birth-weight infants^13,14^, monozygotic twins^15–17^, and ill neonates requiring medication^18,19^ or blood transfusions^20^ may exhibit lower TSH levels at birth, increasing the risk of false-negative results. Furthermore, the chosen TSH cutoff value influences the balance between false positives and false negatives, with lower cutoffs potentially reducing false negatives but increasing the false-positive rate and subsequent recall rate^12,21^. Therefore, exploring alternative CH screening methods, such as genetic testing or the use of additional biomarkers, is warranted to minimize both false-positive and false-negative diagnoses.

Dried blood spots (DBS) represent a well-established medium for clinical diagnostics, serving as the foundation for neonatal metabolic screening for decades^22,23^. Modern applications have significantly expanded the utility of DBS-derived protein and peptide biomarkers to include screening for Wilson’s disease via ATP7B measurement, monitoring primary immunodeficiencies, and assessing population health status during the COVID-19 pandemic^24,25^. These advancements underscore the versatility of DBS as a minimally invasive tool for improving diagnostic accuracy and longitudinal care, particularly as peptide biomarkers have emerged as powerful diagnostic indicators capable of reflecting complex physiological and pathological states^26,27^. Furthermore, while various studies have successfully utilized DBS for the detection of infectious diseases^28–30^, the integration of these biomarkers into broader clinical practice remains a promising frontier for disease detection and therapeutic monitoring.

Among the most promising analytical platforms for biomarker discovery and clinical diagnostics is matrix-assisted laser desorption/ionization time-of-flight mass spectrometry (MALDI-TOF MS). This technology offers a high-throughput, sensitive, and specific approach for analyzing complex biological samples^31^. Its ability to provide rapid peptide profiling has proven highly effective in medical research, particularly for distinguishing cancerous from non-cancerous conditions^32–37^. A notable application is the successful implementation of MALDI-TOF MS for neonatal screening, where it has been used to analyze hemoglobin mass patterns in DBS for the detection of sickle cell disease and thalassemia^38–40^.

Despite these advances, the potential of a comprehensive peptide biomarker approach for the diagnosis of CH remains underexplored. Therefore, this study aims to identify novel peptide barcodes and potential biomarkers in DBS samples from Thai neonates. A MALDI-TOF peptide profile database to differentiate CH-negative and CH-positive individuals will be generated. To gain deeper insights, we will investigate disease-specific peptide profiles using MALDI-TOF MS, complemented by liquid chromatography-tandem mass spectrometry (LC-MS/MS). A unique peptide barcode can serve as an effective CH screening method, and the identified peptide biomarkers will provide insights for diagnosis and guide future personalized treatment strategies.

## Results

### Demographic characteristics of DBS leftover

Table 1 presents the demographic characteristics of the 470 neonates included in the study, comprising 400 CH-negative and 70 CH-positive cases. The CH-negative group consisted of 257 males and 143 females, with gestational ages ranging from 37 to 43 weeks and birth weights from 2,500 to 5,726 g. These neonates initially screened positive for CH in DBS but were subsequently confirmed as negative via follow-up serum testing. The CH-positive group comprised 35 males and 35 females, with gestational ages of 37–41 weeks and birth weights ranging from 2,550 to 4,500 g. All neonates in this group tested positive for CH in both the initial DBS screening and subsequent confirmatory serum testing.

**Table 1:** The demographic characteristics of the 470 neonates included in the study, comprising 400 CH-negative and 70 CH-positive cases.

Sample characteristics	CH Negative	CH Positive *
Sex, n (%)		
Male	257 (64.25)	35 (50)
Female	143 (35.75)	35 (50)
Gestation age, min–max (weeks)	37–43	37–41
Birth weight, min–max (grams)	2,500–5,756	2,550–4,500
Screening test in DBS		
TSH, min–max (mU/L)	25.00–>180.68	25.58–>180.68
Confirmatory test in serum		
TSH, min–max (mU/L)	1.06–9.95	34.97–1,007.36
FT_4_, min–max (ng/dL)	1.02–1.97	0.03–0.82

NOTE: * CH Positive is indicated when the serum levels of TSH and FT_4_ are both above 11 mU/L and below 0.83 ng/dL, respectively.

CH = Congenital hypothyroidism, DBS = Dried blood spot, TSH = Thyroid stimulating hormone, FT_4_ = Free thyroxine, mU/L= Milliunits per liter, ng/dL = Nanograms per deciliter.

### Evaluation of CH based on DBS screening and serum confirmation results

In the CH-negative group (*n* = 400), DBS TSH levels ranged from 25.00 to 49.88 mU/L in 379 neonates and from 50.55 to 180.68 mU/L in 21 neonates. All neonates with DBS TSH ≥ 25.00 mU/L were recalled for serum TSH and FT4 analysis. In these CH-negative neonates, serum TSH ranged from 1.06 to 9.95 mU/L, and serum FT4 ranged from 1.02 to 1.84 ng/L. Among the CH-positive group (*n* = 70), 12 neonates had DBS TSH values between 25.00 and 50.00 mU/L, and 58 neonates had DBS TSH values between 50.00 and 180.68 mU/L; all of these neonates also underwent serum confirmation. Serum TSH in the CH-positive group ranged from 34.97 to 766.62 mU/L, and serum FT4 ranged from 0.03 to 0.82 ng/L. Consistent with diagnostic expectations, serum TSH was lower and serum FT4 remained within the reference range in the CH-negative group, whereas serum TSH was elevated and serum FT4 was decreased in the CH-positive group.

### MALDI-TOF peptide barcoding of congenital hypothyroidism in DBS leftover

**Fig. 1:**
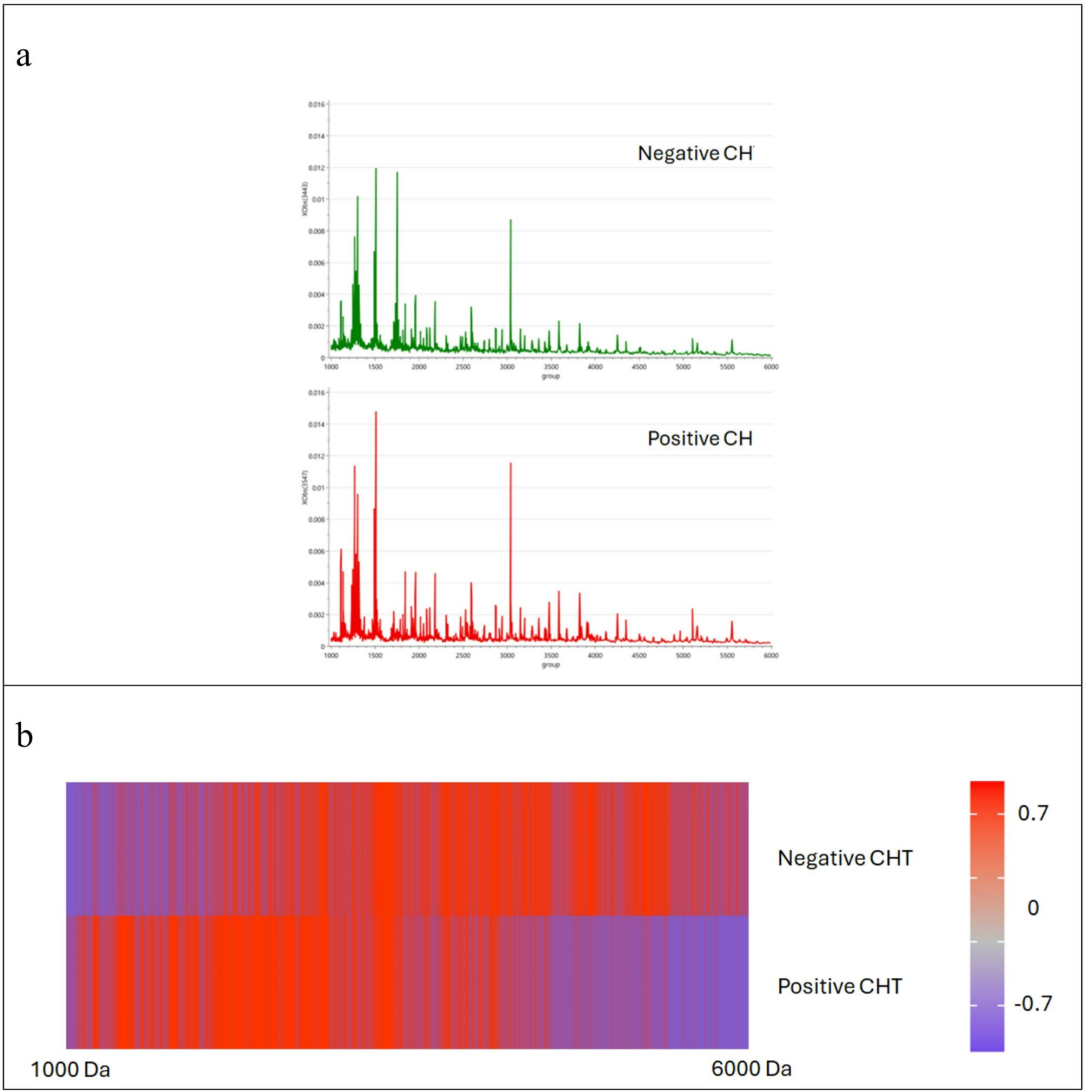
Peptide barcode analysis of CH-negative and CH-positive samples via MALDI-TOF MS. (**a**) Representative MALDI-TOF MS spectra comparing CH-negative and CH-positive patients within the range of 1,000–6,000; a total of 1,434 peptide features were detected. (**b**) Hierarchical heatmap visualization of the peptide barcode. Relative expression levels are represented by color intensity, where red denotes high expression and blue denotes low expression. The heatmap was generated using MetaboAnalyst 6.0^53^(https://www.metaboanalyst.ca/).

A total of 1,434 peptide features were detected in 400 CH-negative and 70 CH-positive samples using MALDI-TOF MS. The peptide barcode within the mass range of m/z 1,000–6,000 Da are depicted in Fig. 1a. The peptide barcode spectra were represented as a heatmap. This visualization, where red indicates high peptide expression and blue indicates low expression at each corresponding m/z position. The results revealed a clear and well-defined separation in peptide patterns across all 470 samples, comprising 400 CH-negative and 70 CH-positive cases (Fig. 1b). This pronounced difference underscores the potential of these peptide profiles as biomarkers for CH.

**Fig. 2:**
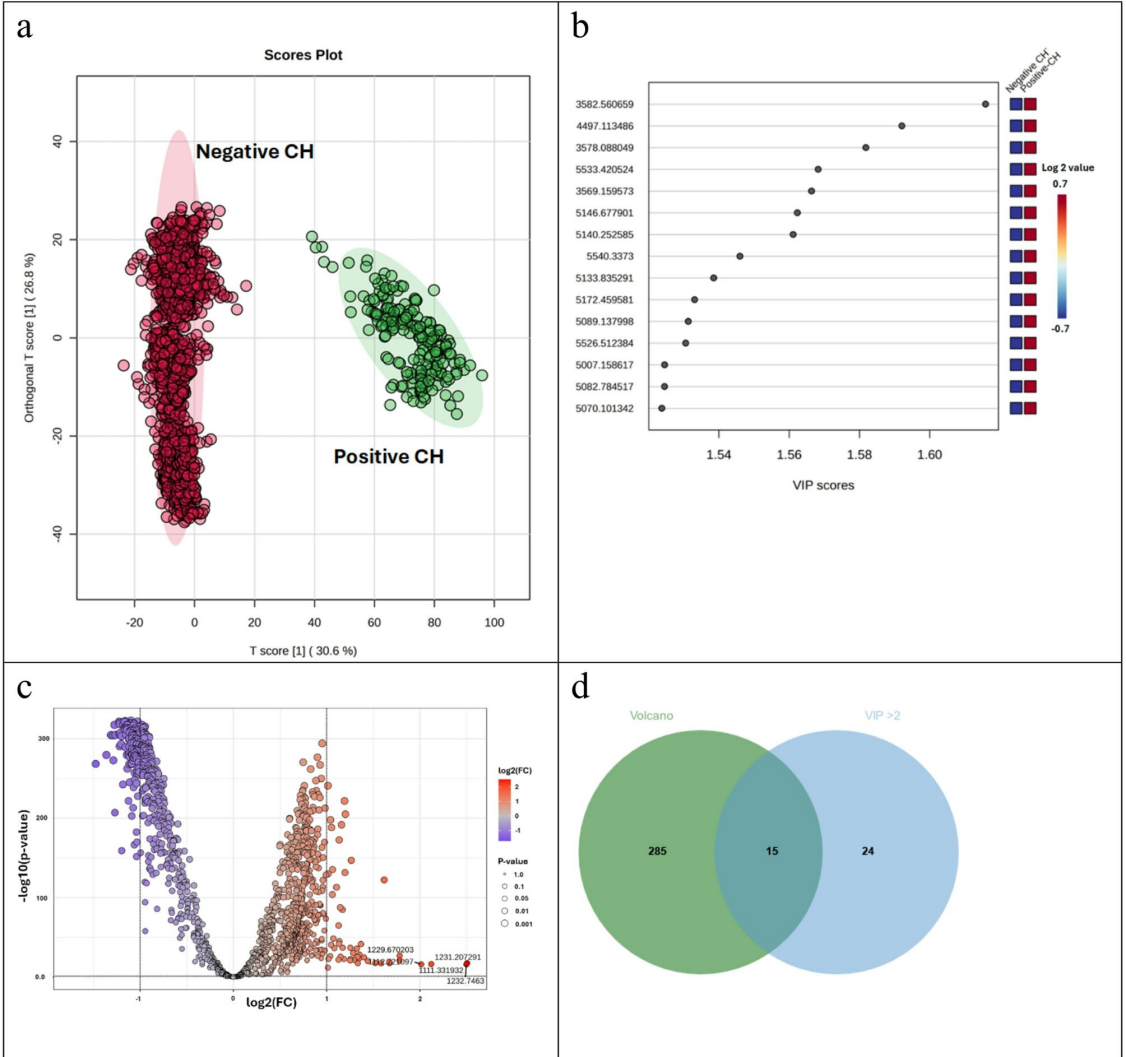
Multivariate analysis and comparison of 1,434 peptide features in CH-negative and CH-positive patient. (**a**) Orthogonal Partial Least Squares Discriminant Analysis (OPLS-DA) demonstrating the distinct classification and separation of the two patient groups. (**b**) PLS-DA VIP score plot ranks the proteins that contribute most significantly to group separation. An accompanying heatmap illustrates the relative abundance of these discriminatory proteins (red, high; blue, low). (**c**) Volcano plot identifies differentially abundant peptides, with those meeting the criteria (≥ 2-fold change and *p* < 0.05) highlighted. (**d**) Venn diagrams displaying the intersection of significantly altered proteins in the CH-negative and CH-positive groups.

Global peptide barcode analysis was performed to identify peptide features capable of distinguishing CH-positive individuals from those with CH-negative. A total of 1,434 peptide peaks derived from MALDI-TOF MS were analyzed using MetaboAnalyst 6.0. To distinguish the classes and to assess the global peptide variations, the peptide profiles from two groups were subjected to statistical analysis using Orthogonal Partial Least Squares Discriminant Analysis (OPLS-DA). OPLS-DA generated a clear separation between positive and negative CH groups (Fig. 2a). Variable Importance in Projection (VIP) scores, a critical metric from the PLS-DA models, were used to evaluate the contribution of individual peptides to the discrimination between experimental groups. The VIP-scored peptides and their corresponding relative expression levels in positive and negative CH groups are depicted in Fig. 2b. Volcano plot analysis identified 300 peptides with significantly altered expression (p-value < 0.05 and fold change > 2) between 2 groups (Fig. 2c).

To refine the list of candidate peptides differentially expressed in positive and negative CH groups, the peptide sets obtained from the PLS-DA VIP scores and Volcano plot analysis were integrated using a Venn diagram (Fig. 2d). This integrated statistical approach applied the following stringent criteria: significant mean differences between groups (Volcano plot: p-value < 0.05 and fold change > 2), high discriminatory power (PLS-DA VIP scores > 2.0). While the integrative analysis identified 15 candidate peptides in total, six of this exhibited unique expression within the CH-positive group and were not detected in the CH-negative samples. These six key peptides are highlighted in the Volcano plot (Fig. 2c) and detailed in Table 2. These corresponded to peptide sequences from key proteins: Q9BXQ6 (Transmembrane protein 121B), Q6P158 (ATP-dependent RNA helicase DHX57), Q8WWM7 (Ataxin-2-like protein), Q701N2 (Keratin-associated protein 5–5), G5E9R7 (Keratin-associated protein 4–16) and O75182 (Paired amphipathic helix protein Sin3b).

**Table 2:** List of six dominant peptides exclusively expressed in the CH-positive patient group, identified by MALDI-TOF peptide barcode analysis.

Peptide mass (Dalton)	Peptide sequence	Uniprot Number	Gene name	Protein name
1,138.25	SGASCCPCCCCC	Q9BXQ6	TMEM121B	Transmembrane protein 121B
1,231.45	WDDGDDFCIF	Q6P158	DHX57	ATP-dependent RNA helicase DHX57
1,232.62	PGPPAAASPCLGPV	Q8WWM7	ATXN2L	Ataxin-2-like protein
1,234.33	CGSGCGGCGGCGSGCA	Q701N2	KRTAP5-5	Keratin-associated protein 5–5
1,242.24	CCESSCCCPCCC	G5E9R7	KRTAP4-16	Keratin-associated protein 4–16
1,249.86	VPVVLKRLKAK	O75182	SIN3B	Paired amphipathic helix protein Sin3b

### Differentially expressed peptides identification by LC-MS

**Fig. 3:**
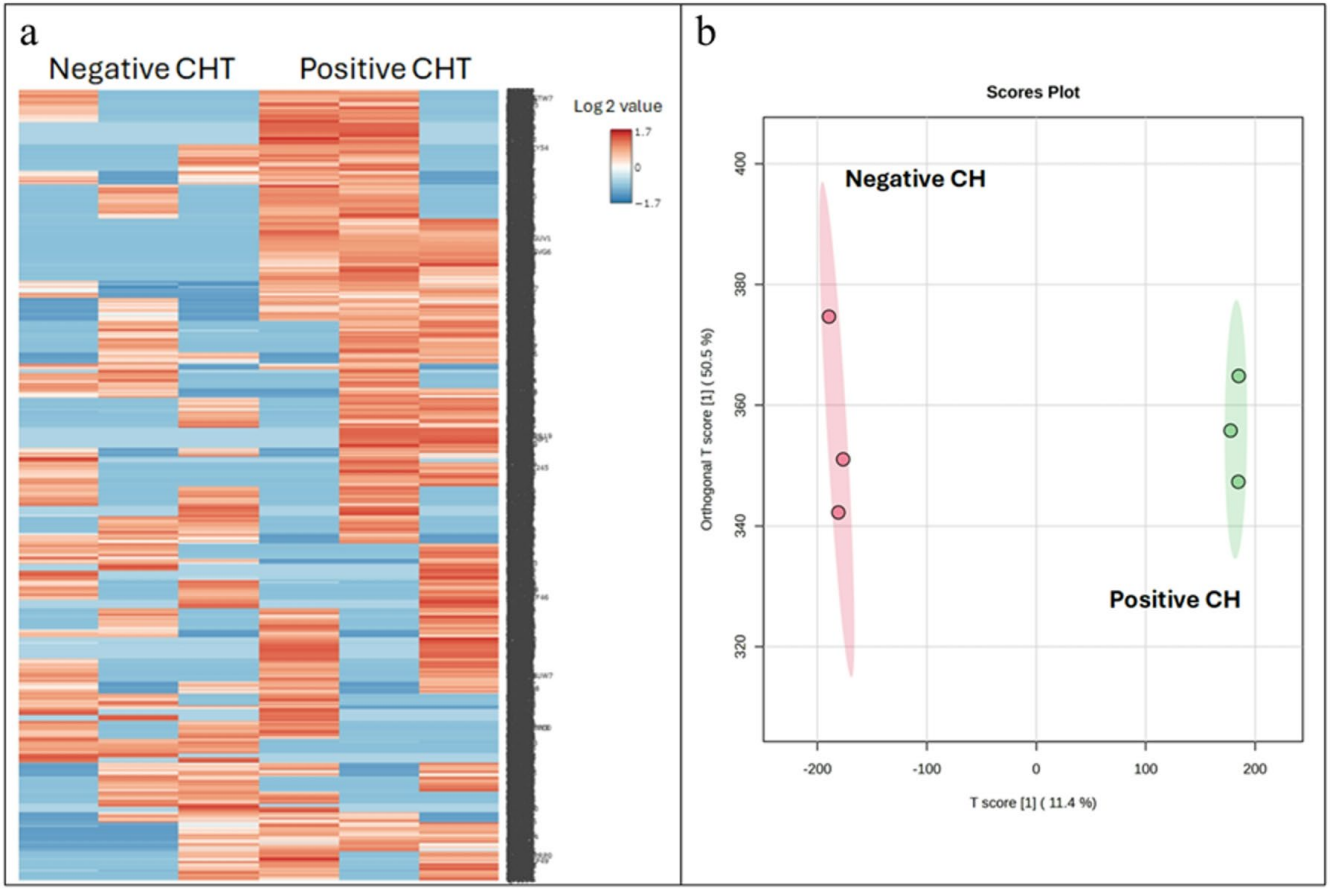
Peptidome profiling of CH-negative and CH-positive samples by LC-MS. (**a**) Heatmap visualization of the relative peptide abundance where red indicates high relative abundance and green indicates low relative abundance. Columns represent individual samples, and rows correspond to peptide sequences. The heatmap was generated using MetaboAnalyst 6.0^53^(https://www.metaboanalyst.ca/). (**b**) Orthogonal Partial Least Squares Discriminant Analysis (OPLS-DA) score plots demonstrating the distinct peptide clustering between CH-negative and CH-positive patients.

**Fig. 4:**
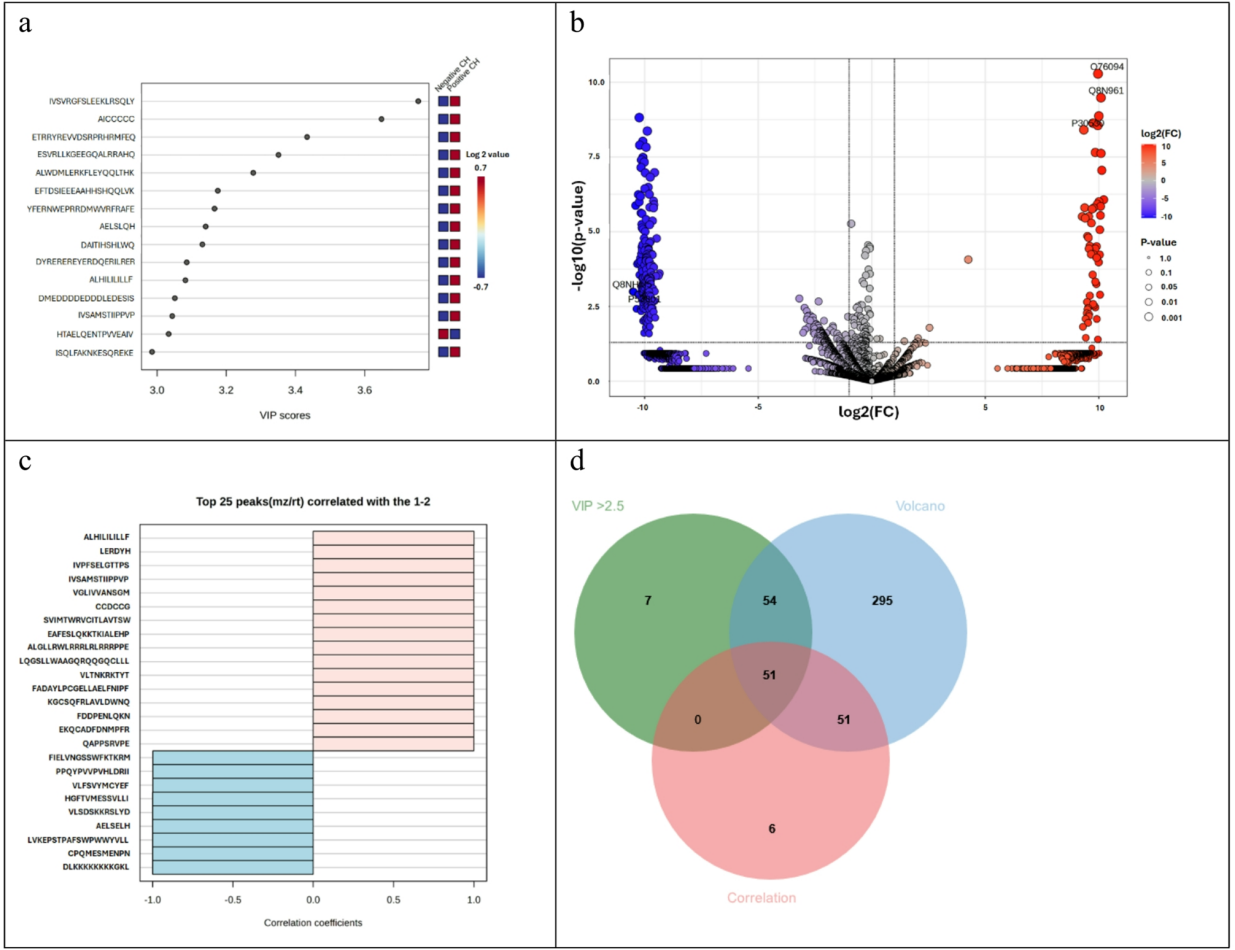
Comparative statistical analysis of peptidomic profiles in CH-negative and CH-positive patients. (**a**) PLS-DA VIP score plot shows proteins driving group separation, with an accompanying heatmap illustrating their relative abundance (red, high; blue, low). (**b**) Volcano plots depict significantly altered peptide abundance between groups (*p* < 0.05, fold change > 2). (**c**) Pearson correlation heatmaps of the top 25 differentially abundant proteins, showing positive (light pink) and negative (light blue) correlations. (**d**) Venn diagrams displaying the overlap of significantly altered peptides identified in both patient groups. Note: Peptides were considered differentially expressed only if they met a consensus of three criteria: Volcano plot (*p* < 0.05, fold change > 2), PLS-DA VIP score (> 2.5), and a strong Pearson correlation (|r|≥0.99).

A label-free-quantitative peptidomics approach was used on peptides extracted from DBS to compare CH-positive and CH-negative samples. Following LC-MS analysis, raw MS/MS data were processed using MaxQuant 2.2.0.0 and searched against Uniprot database (*Homo sapiens*). A total of 11,162 peptides were identified at an FDR of < 1%; their global expression profiles are summarized as a heatmap in Fig. 3A. Subsequent bioinformatic analysis via MetaboAnalyst 6.0 included Orthogonal Partial Least Squares Discriminant Analysis (OPLS-DA), which clearly separated the two CH groups (Fig. 3b). The Variable Importance in Projection (VIP) scores from the OPLS-DA identified the most critical peptides driving this separation; the top 15 discriminatory proteins are summarized in Fig. 4a.

To identify significantly altered peptides, the data were filtered based on t-test results (*p* < 0.05) and a > 2 fold-change threshold. Three hundred twenty-six peptides showed a greater than two-fold change in abundance between the CH-positive and CH-negative samples. The Volcano plot (Fig. 4b) illustrates these changes: 268 peptides were significantly increased and 58 were decreased in the CH-positive group compared to the CH-negative group, while 9,674 peptides showed no significant difference. Further Pearson correlation analysis (using the Pattern Hunter feature) assessed the linear association between the groups, with the top 25 differentially abundant peptides showing positive (light pink) and negative (light blue) correlations (Fig. 4c). To refine the list of candidate peptides for CH, data from the Volcano plot (p-value < 0.05 and fold change > 2), OrthoPLS-DA (VIP score > 2.5), and correlation analysis ([r]: 0.99 ≤ *r* ≤ 1 or -1 ≤ *r* ≤ -0.99) were integrated via a Venn diagram (Fig. 4d). This stringent, integrated statistical approach revealed a final set of 37 candidate peptides distinct between CH-negative and CH-positive patients. All 37 candidate peptides analyzed via LC-MS/MS exhibited unique expression in the CH-positive group and were entirely absent in the CH-negative control group.

### Network analysis of peptides extracted from DBS with Congenital hypothyroidism

**Fig. 5:**
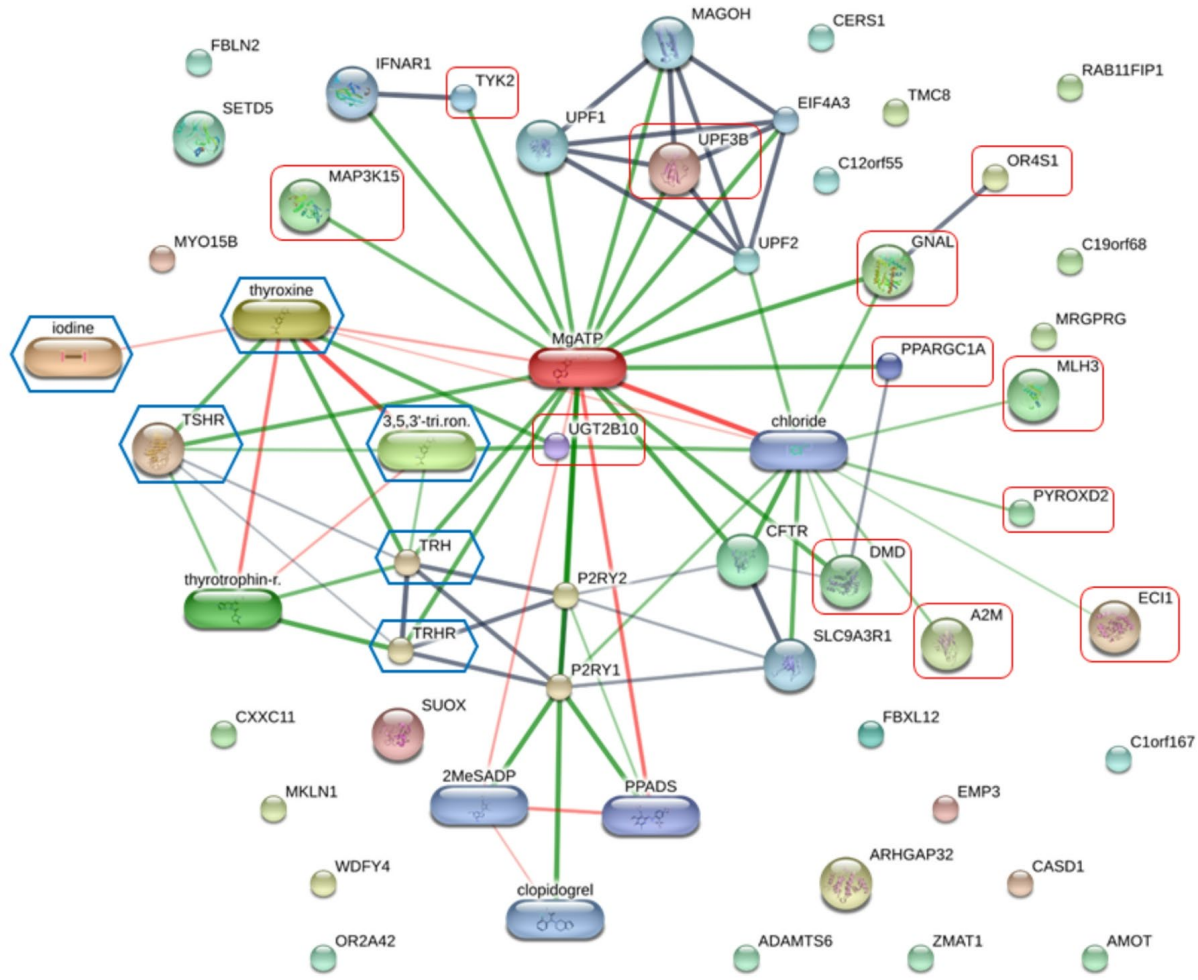
Network of protein-chemical interactions using the STITCH database. Proteins and chemicals are represented as nodes. The line thickness indicates the degree of association. Interactions are color-coded: Gray for protein-protein, green for protein-chemical, and red for chemical-chemical. Proteins identified in this study are highlighted with red squares. Related CH agents (thyroxine, triiodothyronine, iodine, TSHR, TRH, and TRHDE) are shown in hexagonal boxes.

Following the identification of candidate peptides, protein-chemical interactions were analyzed using the STITCH database to investigate potential mechanisms of action. Common chemical and protein relevant to CH, specifically thyroxine, triiodothyronine, iodine, thyroid stimulating hormone receptor (TSHR), thyrotrophin-releasing hormone (TRH), and thyrotropin-releasing hormone degrading enzyme (TRHDE), were integrated into the analysis to predict associations and computational interactions. The resulting network analysis, illustrated in Fig. 5, demonstrated interactions between the identified peptides (12 out of 37 and these CH related agents. Interestingly, direct associations were identified between UDP glucuronosyltransferase 2 family, polypeptide B10 (UGT2B10) peptide and thyroxine, triiodothyronine, alongside indirect interactions involving ECI1, PYROXD2, MLH3, A2M, OR4S1, GNAL, DMD, PPARGC1A, TYK2, MAP3K15, and UPF38 (Table 3).

**Table 3:** List of 12 identified peptides highly expressed in CH-positive patients and their correlation with key CH agents: thyroxine, triiodothyronine, iodine, TSHR, TRH, and TRHDE.

Peptide sequence	Uniprot ID number	STITCH ID number	Protein name
FADAYLPCGELLAELFNIPF	P36537	UGT2B10	UDP-glucuronosyltransferase 2B10
HAHLRVTAA	P01023	A2MG	Alpha-2-macroglobulin
ALIHSHRPDLFDW	P11532	DMD	Dystrophin
AGVAVM	P42126	ECI1	Enoyl-CoA delta isomerase 1, mitochondrial
IVSAMSTIIPPVP	P38405	GNAL	Guanine nucleotide-binding protein G(olf) subunit alpha
SAIEWYRKG	Q6ZN16	MAP3K15	Mitogen-activated protein kinase 15
IVEEFIREQLELLQTTGG	Q9UHC1	MLH3	DNA mismatch repair protein Mlh3
VGLIVVANSGM	Q8NGB4	OR4S1	Olfactory receptor 4S1
LVGEDQPLCPDLPELDLS	Q9UBK2	PPARGC1A	Peroxisome proliferator-activated receptor gamma coactivator 1-alpha
PDLERIFGLPGG	Q8N2H3	PYROXD2	Pyridine nucleotide-disulfide oxidoreductase domain-containing protein 2
LVMSIRDGIHGPLLEPFVQ	P29597	TYK2	Non-receptor tyrosine-protein kinase TYK2
QEEERRRQKER	Q9BZI7	UPF3B	Regulator of nonsense transcripts 3B

## Discussion

Congenital hypothyroidism (CH), a prevalent neonatal endocrine disorder, requires timely diagnosis and intervention to prevent irreversible neurodevelopmental consequences. Although Thailand’s national CH screening program, implemented in 1996 and utilizing TSH measurement in DBS via ELISA, has significantly advanced early detection, inherent limitations of TSH-based screening persist. Factors including sample collection timing, disinfectant use, maternal iodine status, DBS preparation, and patient-specific variables such as prematurity, multiple gestation, neonatal illness, and blood transfusions, coupled with variations in TSH cutoff values, can contribute to both false-positive and false-negative results. Consequently, the investigation of alternative screening methodologies is warranted. MALDI-TOF MS, a high-throughput technology with demonstrated efficacy in newborn screening for other conditions, presents a promising alternative. This study explored the potential of MALDI-TOF MS for CH screening using DBS samples by establishing peptide profile databases from Thai neonates to identify discriminatory biomarkers between CH-positive and CH-negative individuals.

The present study introduces a novel diagnostic approach utilizing DBS peptide barcoding coupled with MALDI-TOF MS to effectively differentiate between CH-positive and CH-negative samples (Fig. 1). The peptide profiles demonstrated a marked and reproducible difference between the CH-positive and CH-negative groups. However, approximately 20% of the detected peptides from volcano plot (Fig. 2c) exhibited significant differential expression was observed. To confirm that this substantial shift reflected genuine biological variation rather than normalization artifacts or systematic biases^41^, an integrated statistical framework was employed. While univariate analyses like volcano plots can be sensitive to scaling-driven shifts in p-values, multivariate Partial Least Squares-Discriminant Analysis (PLS-DA) VIP scores (Fig. 2b) offer greater robustness against compositional bias by identifying features based on their global contribution to class separation^42,43^. By implementing a ‘dual-filter’ approach requiring both statistical significance (Volcano plot: p-value < 0.05 and fold change > 2) and high discriminatory power (PLS-DA VIP scores > 2.0), the risk of results being skewed by a few high-abundance peptides was reduced. The high degree of concordance between (PLS-DA) and univariate (Volcano) results confirms that these changes represent systematic alterations across the peptidome rather than technical outliers. Applying these rigorous selection criteria yielded 15 candidate peptides (Fig. 2d), including six unique markers observed only in CH-positive samples. Specifically, six dominant peptides with m/z values of 1,138.25, 1,231.45, 1,232.62, 1,234.33, 1,242.24, and 1,249.86 demonstrated exclusive presence in the CH-positive group (Table 2). This distinct molecular profile serves as a high-confidence signature for differentiating CH status. This evidence aligns with a growing body of literature supporting the utility of MALDI-TOF MS as a robust, rapid, and cost-effective tool for diagnosis and prognosis in various pathological conditions, particularly malignancies. For instance, Thanasukarn et al. (2025)^37^ successfully utilized MALDI-TOF MS-derived peptide profiles to rapidly discriminate between early and late recurrence in and identified potential serum peptide biomarkers to enhance recurrence classification accuracy. Similarly, serum peptide signatures have been successfully employed for the diagnosis and classification of hepato-pancreato-biliary cancers^34^, and a signature peptide pattern for cervical cancer screening was previously established by Rungkamoltip et al. (2023)^35^. Collectively, these studies underscore that MALDI-TOF MS facilitates the discovery of disease-associated peptides, offering significant potential to improve early screening and diagnosis due to its reproducibility, reduced time consumption, low cost, and high-throughput capability.

Upon obtaining peptide barcode of DBS to distinguish between negative and positive CH patients, MALDI-TOF MS provides rapid screening. The linear TOF’s low resolution and lack of fragmentation limited its initial discriminative power^44^. To resolve this, LC-MS/MS was utilized as a complementary approach, leveraging its superior resolution to definitively identify CH-associated peptide sequences. Because of fundamental differences in ionization and detection, LC-MS/MS was used to provide independent structural identification rather than direct value verification^45^. By combining the high-throughput barcoding of MALDI-TOF MS with the high-confidence identifications from LC-MS/MS, classification accuracy and disease detection were significantly improved. This integrated peptidome-based strategy aligns with successful applications reported in CCA^34,37^ and lung cancer research^46^, demonstrates that merging rapid mass profiling with precise sequence data provides a more robust and precise framework for diagnosing CH in dried blood spots.

To minimize genetic variation and identify representative peptide biomarkers for positive and negative CH, the peptides extracted from each group were pooled in equal peptide amounts and performed peptidome analysis using LC-MS/MS. Pooled peptides were analyzed using LC-MS/MS, identified 11,162 peptides. To evaluate differential peptide expression, Volcano plots (p < 0.05, fold change > 2) was employed to compare peptide profiles between CH-negative and CH-positive group. The certain large fold-changes were driven by ‘zero’ values in the CH-negative group was observed (Fig. 4b), however, these represent peptides that were consistently undetected across all replicates, suggesting high biological specificity rather than stochastic technical noise. To ensure these extreme values did not skew the interpretation, cross-validation of the univariate results (Volcano) with PLS-DA VIP scores and Pearson correlation was performed. These multivariate approaches are inherently more robust to individual fold-change magnitudes, as they prioritize a feature’s overall contribution to group separation and variance^43^. Partial Least Squares Discriminant Analysis (PLS-DA) were implemented to discern peptide profiles between CH-negative and CH-positive group. Peptides exhibiting Variable Importance in Projection (VIP) scores exceeding 2.5 were considered significant contributors to the PLS-DA model (Fig. 4a). Correlation analysis, utilizing the Pattern Hunter feature, was performed to identify peptide expression changes, with peptides ranked by Pearson correlation coefficient (Fig. 4c). Venn diagrams were generated to visualize the overlap and distinctions among peptide lists derived from these different differential analyses (Fig. 4d). This integrative approach identified 37 candidate peptides, subsequent network analysis demonstrated interactions between a subset of these identified peptides (12 out of 37) and CH related agents, namely thyroxine, triiodothyronine, iodine, thyroid stimulating hormone receptor (TSHR), thyrotrophin-releasing hormone (TRH), and thyrotropin-releasing hormone degrading enzyme (TRHDE), as depicted in Fig. 5. The essential components of the hypothalamic-pituitary-thyroid axis were also incorporated into the analysis due to their homeostatic role. This axis operates through a classic negative feedback loop: TRH stimulates TSH release, which drives thyroid hormone (T3 and T4) production. These hormones subsequently inhibit TRH and TSH secretion. The breakdown of TRH, a key homeostatic step, is mediated by TRH-degrading enzyme (TRH-DE). The STITCH network analysis facilitated the functional exploration of peptide-chemical and peptide-peptide interactions, offering insight into potential biological pathways and disease progression dynamics. These findings support the utility of these peptides for developing future peptide-based diagnostic assays.

Peptide biomarkers are highly specific amino acid chains that provide crucial diagnostic information on disease progression and therapeutic response. Their ability to reflect molecular-level changes has driven their established use in clinical practice, exemplified by PSA^22^ and CEA/CA 19 − 9^47^. Peptidomics has proven particularly effective in oncology for cancer detection and monitoring^36,48^. Here, the network analysis in Fig. 5 connects the 12 identified proteins to CH-related agents. UGT2B10 demonstrated a direct association with thyroxine and triiodothyronine, while the other 11 proteins (e.g., MAP3K15, ECI1, PYROXD2) were involved in indirect interactions. This association suggests these peptides could serve as specific peptide biomarkers for CH, highlighting the substantial promise of peptidomics in advancing personalized medicine for this condition.

Because the peptidome comprises both proteolytic degradation products of larger proteins and peptides directly encoded by the genome^49,50^, identifying both the specific sequence and its protein of origin is therefore essential. This dual characterization provides a comprehensive view of the biological processes and cleavage events associated with CH status. By mapping these peptides to their parental proteins can infer changes in protease activity and protein turnover that may otherwise remain undetected at the total protein level. Consequently, this approach allows for the identification of specific ‘cleavage signatures’ that serve as sensitive indicators of the physiological shifts occurring within the CH-positive group.

Limitations of the current study underscore the need for cautious interpretation. To confirm and strengthen the clinical applicability and generalizability of our findings, future research must involve multicenter investigations with larger, more diverse populations and independent external validation cohorts. In parallel, the promising peptides identified here will undergo LC-MS/MS identification. This step is crucial for developing a robust biomarker panel, which is the ultimate goal of MS-based peptidome research for clinical translation.

In conclusion, current TSH-based screening for CH is hindered by numerous limitations that impact diagnostic accuracy. A novel, high-throughput diagnostic approach using MALDI-TOF MS peptide barcoding of DBS that effectively and reproducibly discriminates between CH was developed, through subsequent LC-MS/MS and network analysis, 12 candidate peptides showed associations to CH-related agents, suggesting their potential as specific peptide biomarkers. This method offers a robust and cost-effective alternative to current screening, representing a significant step toward improving early CH detection and advancing personalized medicine. Future multicenter validation and peptide identification are essential for clinical translation.

## Methods

### Study groups

Residual DBS samples from CH screenings conducted between 2020 and 2021 at the Neonatal Screening Operation Centre, Department of Medical Sciences, Ministry of Public Health, Nonthaburi, Thailand, were used for MALDI-TOF MS analysis. The study population consisted of Thai neonates aged 2–7 days at the time of DBS collection (heel prick or dorsal hand vein venipuncture), all of whom were born at ≥ 37 weeks gestation with birth weights ≥ 2,500 g. Inclusion criteria were: (1) absence of recorded neonatal diseases or abnormalities such as pneumonia, early-onset sepsis (EOS), respiratory distress syndrome (RDS), transient tachypnea of the newborn (TTNB), or meconium aspiration syndrome (MAS), and no administration of antibiotics (e.g., ampicillin, penicillin, gentamicin, cloxacillin, or cefotaxime) or other medications; (2) intact DBS samples with fully saturated blood spots permeating to the reverse side of the filter paper, free from microbial or chemical contamination; and (3) confirmatory serum testing for TSH (reference range: 0.72–11 mU/L) and free T4 (FT4) (reference range: 0.83–3.09 ng/dL)^8^ to classify neonates as either CH-positive or CH-negative. DBS samples exhibiting incomplete blood spot saturation (i.e., visible white areas within the circle) or evidence of microbial or chemical contamination were excluded.

The experimental research was conducted in January 2022. The study protocol was approved by the Human Research Ethics Committee of the Department of Medical Sciences, Ministry of Public Health, Nonthaburi, Thailand, on June 8, 2021 (Project No. 8/2564). All experiments were performed in accordance with the international guidelines for human research protection as Declaration of Helsinki, The Belmont Report, CIOMS Guideline, International conference on Harmonization in Good Clinical Practice (ICH-GCP) and 45 CFR 46.101(b). This study utilized fully anonymized ‘leftover’ clinical samples. As the research involved only secondary analysis of existing biological materials and required no new interventions or patient contact, it was classified as posing minimal risk. Accordingly, the Department of Medical Sciences Research Ethics Committee (DMSc REC), Department of Medical Sciences, Ministry of Public Health, Thailand granted a waiver of informed consent for this study.

### Determination of TSH in Dried Blood Spot

Neonatal screening for CH was performed by measuring TSH levels in DBS samples using an in-house sandwich ELISA, as previously described by Charoensiriwatana et al. (2003)^7^. Briefly, TSH was extracted by punching 1/8-inch diameter circles from DBS samples and TSH calibrator cards (equivalent to a serum volume of 1.37–1.71 µL) into wells of a 96-well microtiter plate pre-coated with mouse anti-TSH. One hundred fifty microliters of buffer were added to each well to elute the blood from the filter paper. Plates were then incubated in a sealed humidity-controlled container at room temperature for 16–18 h. Following incubation, the supernatant and filter paper were removed, and the plates were washed once with 0.9% normal saline. TSH levels were subsequently determined using an automated colorimetric immunoassay analyzer (Explorer™ G3 Workstation; PerkinElmer, USA).

The assay procedure continued with three washes using 300 µL of wash buffer. One hundred microliters of anti-TSH peroxidase conjugate solution were then added to each well, and the plate was incubated for 100 min at room temperature. After a further three washes, 150 µL of substrate solution was added, and the plate was incubated in the dark for 20 min. The reaction was terminated by the addition of 50 µL of stop solution. Absorbance was measured at 450/620 nm, and data were analyzed using Gen5 3.09 software (Agilent Technologies, USA). A standard curve was generated from the TSH calibrator data, and sample TSH concentrations were determined by interpolation from this curve, thus reflecting equivalent serum TSH levels. A TSH concentration ≥ 25 mU/L was considered a presumptive positive result for CH screening, while TSH values < 25 mU/L were classified as negative.

### Determination of serum TSH and Free T4

All DBS samples exhibiting elevated TSH levels (≥ 25 mU/L) were further evaluated for CH by measuring serum TSH and FT4 concentrations. Serum TSH was quantified using an in-house ELISA, following the previously described protocol for the DBS assay, with the exception of omitting the sample extraction step. Reference ranges for normal serum TSH and FT4 levels were 0.72–11 mU/L and 0.83–3.09 ng/dL, respectively.

Serum TSH levels were measured manually using 50 µL aliquots of serum calibrators and neonatal samples. Serum free thyroxine (FT4) levels were determined by competitive ELISA using the Monobind AccuBind ELISA Microwells FT4 kit (Monobind Inc., USA), strictly adhering to the manufacturer’s protocol without modifications. Briefly, 50 µL of serum references and samples were added to wells of a 96-well microtiter plate pre-coated with anti-thyroxine antibody. Subsequently, 100 µL of anti-thyroxine peroxidase conjugate solution was added to each well. Following gentle mixing, the plate was incubated for 60 min at room temperature. After incubation, the plate was washed three times with 350 µL of wash buffer. One hundred microliters of substrate solution were then added, and the plate was incubated in the dark for 15 min. The reaction was terminated by the addition of 50 µL of stop solution. Absorbance was measured at 450/620 nm using a microplate reader. FT4 concentrations in the serum samples were determined by comparing their absorbance values to the generated standard curve. Reference ranges for normal newborn TSH and FT4 levels are 0.72–11 mU/L and 0.83–3.09 ng/dL, respectively^8^.

### Sample preparation for mass spectrometric analysis

DBS samples from neonates, previously categorized as CH positive or negative, were punched into 1/8-inch diameter circles. Each punched circle was then immersed in 200 µL of 5% trifluoroacetic acid (TFA) in acetonitrile and agitated at 2,500 RPM for 30 min. Following this extraction step, the resulting peptide samples were collected for subsequent analysis by MALDI-TOF MS and LC-MS.

### Peptide barcode analysis using MALDI-TOF MS

Peptide concentration in each sample was determined using the Bradford assay (Bio-Rad) with bovine serum albumin (BSA; Sigma-Aldrich) as a standard, following the manufacturer’s instructions. For MALDI-TOF MS analysis, peptide samples were prepared by mixing 1 µL of sample with 1 µL of matrix solution. The matrix solution consisted of 10 mg/mL 4-chloro-α-cyanocinnamic acid (4-CCA; Sigma-Aldrich) dissolved in 50% acetonitrile (ACN; Merck) and 0.5% trifluoroacetic acid (TFA; Sigma-Aldrich). One microliter of the resulting mixture was then deposited onto a 384-well ground steel MALDI target (MTP 384, JEOL Ltd., Japan) and allowed to air-dry at ambient temperature.

MALDI-TOF MS spectra were acquired using a JMS-S3000 SpiralTOF mass spectrometer (JEOL Ltd., Japan) operating in linear positive-ion mode. Spectra were collected across a mass-to-charge (m/z) range of 1,000–6,000 Da. Five hundred laser shots at a frequency of 50 Hz were accumulated per sample. Data acquisition was performed using JEOL msTornado Control software (version 1.16, JEOL, Japan). Spectral processing, including smoothing, variance stabilization, baseline correction, and peak detection, was conducted using JEOL msTornado Analysis software (version 1.15, JEOL, Japan) with default parameters. Processed spectra were subsequently exported in comma-separated value (CSV) format. Mass binning was performed at 1.0 Da intervals across an extended m/z range of 1,000–10,000 Da. Prior to sample analysis, external mass calibration was performed in positive-ion mode using a suite of reference peptides with known m/z values: Angiotensin II (m/z 1,046), P14R (m/z 1,533), human ACTH fragment 18–39 (m/z 2,465), bovine insulin oxidized B chain (m/z 3,465), and bovine insulin (m/z 5,731). Instrument calibration was achieved by manual peak assignment within JEOL msTornado Control software (version 1.16) using the reference peptide list, resulting in a mass accuracy of ± 100 ppm.

### Peptidome analysis using LC-MS

Peptide samples were analyzed using an Ultimate 3000 Nano/Capillary LC System (Thermo Fisher Scientific, UK) coupled to a Hybrid quadrupole Q-TOF impact II™ mass spectrometer (Bruker Daltonics) equipped with a Nano-captive spray ion source. One microliter of each peptide digest was loaded onto a µ-Precolumn (300 μm i.d. x 5 mm, C18 Pepmap 100, 5 μm, 100 Å; Thermo Fisher Scientific, UK) for on-line desalting and preconcentration. Peptide separation was performed on a 75 μm I.D. x 15 cm analytical column packed with Acclaim PepMap RSLC C18, 2 μm, 100 Å, nanoViper (Thermo Fisher Scientific, UK), maintained at 60 °C. Mobile phases consisted of 0.1% formic acid in water (Solvent A) and 0.1% formic acid in 80% acetonitrile (Solvent B). Peptides were eluted using a linear gradient of 5–55% Solvent B over 30 min at a constant flow rate of 0.30 µL/min.

Electrospray ionization was performed using the CaptiveSpray source at a voltage of 1.6 kV. Nitrogen was employed as the drying gas at a flow rate of approximately 50 L/h. Collision-induced dissociation (CID) was performed using nitrogen as the collision gas. Mass spectra (MS) and MS/MS spectra were acquired in positive-ion mode at a frequency of 2 Hz over the mass-to-charge (m/z) range of 150–2,200. The collision energy was optimized to 10 eV as a function of m/z. Each sample was analyzed in triplicate to ensure reproducibility.

### Bioinformatics analysis of peptidomics data

Peptide identification and label-free quantification utilized MaxQuant software (version 2.2.0.0)^51^ with the Andromeda search engine. The MS/MS spectra were searched against the UniProt *Homo sapiens* database with the enzyme specificity set to unspecific digestion. Variable modifications were set for methionine oxidation and N-terminal protein acetylation. To improve identification across samples, matching between runs was enabled. The protein false discovery rate (FDR) was set to 1% based on reverse-sequence searches, with a maximum of five modifications permitted per peptide. All other parameters remained at the standard settings.

The MaxQuant ProteinGroups.txt output was imported into Perseus software (version 2.2.0.0)^52^ for downstream analysis. Contaminants were first removed. Protein intensities were log2-transformed, and missing values were imputed using a constant value of zero. Pairwise comparisons between experimental conditions were conducted using Student’s t-tests. MetaboAnalyst^53^ was utilized to visualize peptide barcodes through heatmaps, providing a global overview of expression profiles. OPLS-DA was employed to maximize class discrimination, while volcano plots were used to identify differentially expressed peptides reaching statistical significance (p-value < 0.05 and fold change > 2). To further resolve metabolic signatures, pattern hunter was applied to identify peptides exhibiting distinct expression correlations. Finally, functional interaction networks were analyzed using the STITCH database (version 5)^54^.

### Data analyses

Statistical analyses were performed using GraphPad Prism (version 6.0 for Windows; GraphPad Software, San Diego, CA, USA) and Microsoft Excel 2013. A *p*-value of less than 0.05 (*p* < 0.05) was considered statistically significant.

## Data Availability

All relevant data are within the manuscript and it supporting information files. The raw MS/MS spectral data are available on the ProteomeXchange repository under registration numbers JPST004103 (https://repository.jpostdb.org/preview/111369978068defe7433599; Access key:9352) and PXD069100.
